# Effect of cytisine on some brain and hepatic biochemical parameters in spontaneously hypertensive rats

**DOI:** 10.2478/v10102-010-0004-4

**Published:** 2010-03-29

**Authors:** Rumyana Simeonova, Vessela Vitcheva, Mitka Mitcheva

**Affiliations:** Laboratory of “Drug metabolism and drug toxicity”, Department of Pharmacology and Toxicology, Faculty of Pharmacy, Medical University, Sofia, Bulgaria

**Keywords:** SHR, cytisine, toxicity, metabolism

## Abstract

Tobacco smoking is a risk factor for variety of cardio-vascular diseases, such as hypertension, myocardial infarction, stroke and many others. It is of great importance for hypertensive patients to stop smoking. One of the medicines widely used for smoking cessation in Bulgaria is the original Bulgarian product Tabex®, which is developed on the basis of natural plant alkaloid cytisine. The aim of the following study was to ivestigate the effects of cytisine on some brain and hepatic biochemical parameters in spontaneously hypertensive rats (SHR), an widely used rodent model for human essential hypertension, and to compare the obtained results with their age-matched normotensive controls Wistar Kyoto (WKY). Multiple cytisine administration did not affect the activity of ethylmorphine-N-demethylase (EMND) and anylinehydroxylase (AH), as well as the quantity of cytochrome P 450, nor in WKY neither in SHR In the liver cytisine increased the MDA quantity both in SHR and in WKY, by 25% (*p*<0.05) and by 29% (*p*<0.05) respectively, while the GSH level was not significantly changed by the compound in both strains. In contrast, on the brain level, cytisine administration to SHR caused more prominent toxicity, resulted in GSH depletion and increased MDA quantity, while in WKY strain did not exert any toxicity. Cytisine did not significantly affect ALAT and ASAT activity in both strains. In conclusion, the results of our study suggest higher brain toxicity of cytisine in spontaneously hypertensive rats, that might be due to their pathophysiological characteristics.

## Introduction

According to the World Health Organisation, one third of the adult population smokes. Epidemiological data show that tobacco smoking provokes many diseases in cardio-vascular, pulmonary and other systems and it is one of the major causes for premature death in the world. Tobacco may aggravate the hypertensive state in patients with cardio-vascular diseases. That is why smoking cessation is obligatory condition for them. Because of the great importance of tobacco dependence, there are many approaches to resolve this problem. The current most effective method for treatment of nicotine addiction is nicotine replacement therapy. In Bulgaria, the most used method is the therapy with the original Bulgarian drug Tabex^®^. The product is developed on the basis of cytisine. Cytisine is an alkaloid from the plant *Laburnum anagyroides Med*., (*Cytisus laburnum* L., Fabaceae) which if widely distributed in the Sought part of Central and Eastern Europe. All parts of this plant contain the alkaloid cytisine, but the largest quantity (up to 3%) is found in seeds. (Tzankova and Danchev, 2007). In Bulgaria, cytisine, as a smoking cessation aid, has been used since the 1960s and has been manufactured and marketed since 1964 as Tabex^®^ (Sopharma, Bulgaria).

Taking into consideration a number of hypertensive patients who smoke and the necessity to stop this vice, paucity of available data on drug metabolism and drug toxicity of cytisine on this pathological state, it is important to characterize the effects of cytisine on some liver and brain biochemical parameters in spontaneously hypertensive rats. SHRs are a suitable model for investigation not only of the cardio-vascular diseases, but also of drug metabolism and drug toxicity in this pathological condition. At the same time, it is well known that SHRs are more prone to liver and brain injury, provoked by some compounds.

The aim of the following study was to investigate the effects of cytisine, administered to SHR for 14 days on some brain and hepatic biochemical parameters.

## Materials and methods

### Reagents and chemicals

All reagents used were of analytical grade. Cytisine was provided by Sopharma Pharmaceuticals, Sofia, Bulgaria. The other chemicals: NaHCO_3_, KH_2_PO_4_, Trichloracetic acid, 2-Thyobarbituric acid, CH_3_COOH, Glucoso-6- phosphate, Semicarbazide, Nicotinamide, Ba(OH)_2_, ZnSO_4_, Ethylmorphine, Anyline, Na_2_S_2_O_5_, NADP, Phenol were purchased form Sigma Chemical Co. (Germany). 2,2′-dinitro-5,5′dithiodibenzoic acid (DTNB), K_2_HPO_4_ and NaH_2_PO_4_×2H_2_O were obtained from MERCK (Germany).

For the aim of the experiments, cytisine was dissolved *ex tempore* in distilled water in quantity for receiving working solutions. The solutions were administered once daily via stomach tube (1 ml/100 g b.w.).

### Animals

Experiments were performed in 12 male SHR (body weight 180–230 g) and 12 WKY (body weight 200–250 g), obtained from Charles River Laboratories (Sulzfeld, Germany). The animals were housed in Plexiglas cages (3 per cage) at 20±2 °C and 12-h light: 12-h dark cycle. Food and water were provided *ad libitum*.

Blood pressure was measured in conscious animals using a semi-automated tail-cuff device (LE5002, Letica S/A, Spain). Before the experimental period, the rats were conditioned to the restraining cylinders and blood pressure measurement. Rats were pre-warmed for 10 min using a temperature – controlled warming holder (37 °C) to facilitate tail blood flow before their blood pressure was measured. The mean of tree tail-cuff readings was used as the systolic and diastolic blood pressure value. Body weight and organ weight (livers and brains) were measured with a standard laboratory scale.

All procedures were approved by the Institutional Animal Care Committee and performed strictly following the principles stated in the European Convention for the Protection of Vertebrate Animals used for Experimental and other Scientific Purposes (ETS 123) (1991).

### Design of the experiment

The animals were divided into four groups (n=6 each). The first group was SHR receiving cytisine at dose 5 mg/kg (Tabex. Product monograph, Sopharma, 2006) p.o., once daily for 14 days. The second group was WKY, receiving cytisine 5 mg/kg po once a day for 14 days. The third and fourth groups included respective controls, untreated animals from two rat strains, which were involved in the experiment from the very beginning and housed under the same standard laboratory conditions as treated animals.

All animals were fasted overnight before euthanasia. 24 hours after the last administered dose of cytisine, the animals of all groups was weighed, euthanized by decapitation and blood, livers and brains were taken for biochemical assessment.

### Preparation of liver microsomes for biochemical assay (Guengerich, 1987)

Rats were decapitated and the livers were excised, perfused with 0.15 M KCl and minced. The latter was homogenized with 3 volumes of 1.17% KCl solution in a glass homogenizer. The liver homogenates were then centrifugated at 10,000 × g for 30 min. The supernatant fractions were centrifugated at 105,000 × g for 60 min. The resulting microsomal pellets were stored at −20 °C until assayed.

### Evaluation of Phase I of biotransformation

#### Assay of aniline 4-hydroxilase activity (Cohen et al., 2000)

4-hydroxilation of aniline to 4-aminophenol, that is chemically converted to a phenol-indophenol complex with an absorption maximum at 630 nm. Enzyme activity is expressed as nmol/min/mg.

#### Assay of EMND activity (Cohen et al., 2000)

The enzyme activity was evaluated by the formation of formaldehyde, trapped in the solution as semicarbazone and measured by the colorimetric procedure of Nash, at 415 nm. Enzyme activity is expressed as nmol/min/mg.

#### Assessment of cytochrome P450 quantity (Omura and Sato, 1964)

At the day of assay the microsomal pellets were resuspended and diluted in phosphate buffer + EDTA (pH=7.4). Liver protein concentration was mesured, using the method of Lowry (1951) and was adjusted to 10 mg/ml. Cyt P450 quantity was quantified spectrophotometrically as a complex with CO, at 450 nm

### Preparation of brain and liver homogenate for MDA assessment (Deby and Goutier, 1990)

Samples of liver and brain tissue were homogenized in 25% trichloracetic acid (TCA) and 0.67% thiobarbituric acid (TBA) in Glass homogenizer (PX-OX 2000). The samples were then mixed thoroughly, heated for 20 min in a boiling water bath, cooled and centrifuged at 4,000 rpm for 20 min. The absorbance of supernatant was measured at 535 nm against a blank that contained all the reagents except the tissue homogenate. MDA level was expressed as nmol/g wet tissue.

### Preparation of brain and liver homogenate for GSH assessment (Fau *et al*., 1994)

Samples of liver and brain tissue were homogenated in 5% trichloracetic acid (TCA) in Glass homogenizer (PX-OX 2000) and centrifugated for 20 minutes at 4,000 rpm.

GSH was assessed by measuring non-protein sulfhydryls after precipitation of proteins with TCA, followed by measurement of thiols in the supernatant by the DTNB reagent (Bump *et al*., 1983). GSH level was expressed as nmol/g wet tissue.

### Evaluation of transaminase activity (ALAT and ASAT) in serum

The blood was taken into a tube containing EGTA. Serum was separated by centrifugation in bench centrifuge (Eppendorf, MiniPlus) at 10,000 rpm for 10 min, 4 °C and serum activity of aspartate aminotransferase (ASAT) and alanine aminotransferase (ALAT), were measured using automated, optimized spectrophotometrical method (COBOS Integra 400 plus, Roche Diagnostics).

### Statistical analysis

The results were presented as ± SD of 6 animals in each group. Student's t-test was used. Probability values less than 0.05 were considered significant.

## Results

### Effect of cytisine on P 450 quantity and enzyme activities (Table 1).

Untreated SHRs presented higher (*p*<0.05) activity of EMND than untreated WKY. Cytisine treatment did not affect the activity of EMND and AH, as well as the quantity of P 450, nor in WKY neither in SHR.

**Table 1:** Effect of cytisine on cytochrome P450 quantity, AH and EMND activity after multiple administration in male SHR, compared to male WKY.

	WKY		SHR	
		
Parameter	Control	Cytisine	Control	Cytisine
**P-450 quantity** (nmol/mg)	0.336 ± 0.002	0.356 ± 0.004	0.365 ± 0.01	0.350 ± 0.009
**AH activity** (nmol/mg/min)	0.043 ± 0.002	0.044 ± 0.003	0.041 ± 0.004	0.036 ± 0.005
**EMND activity** (nmol/mg/min)	0.332 ± 0.007	0.305 ± 0.011	0.472 ± 0.04^*^	0.511 ± 0.02

Data are expressed as mean ± SD of 6 rats,

^*^ *p*<0.05 vs control WKY

### Effect of cytisine on hepatic GSH and MDA levels (Table 2)

Untreated SHRs showed lower (by 26% (*p*<0.05) GSH level and higher (by 38% (*p*<0.05) MDA quantity than WKY untreated group. Cytisine increased the MDA quantity both in SHR and in WKY, by 25% (*p*<0.05) and by 29% (*p*<0.05) respectively, while the GSH level was not significantly changed by the compound in both strains.

**Table 2:** Effect of cytisine multiple administrations on hepatic GSH level and MDA quantity in male SHR, compared to male WKY.

	WKY		SHR	
		
Parameter	Control	Cytisine	Control	Cytisine
**GSH** (nmol/g)	6.34 ± 0.16	5.74 ± 0.16	4.69 ± 0.14^*^	4.47 ± 0.16
**MDA** (nmol/g)	1.05 ± 0.03	1.35 ± 0.11^*^	1.45 ± 0.04^*^	1.81 ± 0.12^+^

Data are expressed as mean ± SD of 6 rats,

^*^ *p*<0.05 vs control WKY;

^+^p<0.05 vs control SHR

### Effect of cytisine on brain GSH and MDA levels (Table 3)

Brains from control SHR group displayed lower GSH level by 30% (*p*<0.05) and higher MDA level by 25% (*p*<0.05), compared to the control WKY group. In brain homogenate from SHR, cytisine decreased GSH level by 25% (*p*<0.05) and increased MDA quantity by 22% (*p*<0.05). The results were compared to the unntreated SHRs. Cytisine did not change these parameters in WKY.

**Table 3:** Effect of cytisine multiple administrations on brain GSH level and MDA quantity in male SHR, compared to male WKY.

	WKY		SHR	
		
Parameter	Control	Cytisine	Control	Cytisine
**GSH** (nmol/g)	1.28 ± 0.03	1.16 ± 0.08	0.89 ± 0.05^*^	0.67 ± 0.07^+^
**MDA** (nmol/g)	3.89 ± 0.05	3.82 ± 0.09	4.86 ± 0.04^*^	5.95 ± 0.07^+^

Data are expressed as mean ± SD of 6 rats,

^*^ *p*<0.05 vs control WKY;

^+^p<0.05 vs control SHR

### Effect of cytisine on transaminases (Table 4)

Cytisine did not affect significantly ALAT and ASAT activity in both strains.

**Table 4:** Effect of multiple administration of cytisine on serum ALAT and ASAT activity in male SHR, compared to male WKY.

	WKY		SHR	
		
Parameter	Control	Cytisine	Control	Cytisine
**ALAT** UI/L	66.2 ± 6.2	61.3 ± 19.1	82.6 ± 4.2	92.7 ± 23.2
**ASAT** UI/L	234.2 ± 12.5	228.3 ± 9.3	329.3 ± 9.8^*^	289.2 ± 22.5

Data are expressed as mean ± SD of 6 rats,

^*^ *p*<0.05 vs control WKY

## Discussion

Cytisine, a natural plant alkaloid, has been used in Bulgaria for 40 years in the clinical management of smoking cessation and has shown similar to nicotine pharmacological characteristics. Being a competitive blocker of α4β2* nAChRs, cytisine behaves as an antagonist in the presence of nicotine and therefore would decrease craving and attenuate nicotine withdrawal symptoms in humans (Lukas, 2006).

Cytisine is considered to be efficient and safe drug for smoking cessation (Etter, 2008). However there is not sufficient clinical experience with Tabex^®^ in patients with chronic diseases, like hypertension, diabetes etc.

Regarding the lack of experimental data about cytisine metabolism and toxicity, in the present study, we investigated the effect of cytisine on some hepatic and brain biochemical parameters, using spontaneously hypertensive rats (SHR), that are considered to be a suitable pathophysiological model for essential hypertension in human (Yoshimoto *et al*. 2003)

Our results on the level of drug metabolizing enzyme systems reveiled higher EMND activity, marker of CYP 3A (Amacher and Schomaker, 1998) in SHR with no difference in cytochrome P 450 quantity and AH activity, marker of CYP 2E1 (Monostory *et al*., 2004), compared to control WKY rats (Table 1). These results are in good correlation with the studies, carried out by Merrick *et al*. (1985) that showed a significant increase in EMND activity in SHR with only slight increases in cytochrome P450 quantity and AH activity, compared to WKY rats. Few years latter Basu *et al*. (1994) proved that this elevated CYP 3A activity in SHR is related to the augmentation of the arterial blood pressure.

14 days oral administration of cytisine did not change the activity of EMND and the activity of AH, as well as the quantity of cytochrome P 450 nor in SHR, neither in WKY. These results are supported by data in the literature that discuss cytisine minimal hepatic biotransformation in humans and 90–95% excretion of the unchanged compound in the urine (Tabex. Product monograph, Sopharma, 2006).

One of the essential factors for hypertension is the oxidative stress, which is characterized by low level of the intracellular protector GSH and high level of MDA, a product of lipid peroxidation and marker of oxidative stress. In their study Cediel *et al*. (2003) proved that in liver homogenates from SHR, MDA levels were higher and the ratio reduced/oxidized glutathione (GSH/GSSG) and glutathione peroxidase activity (GPx), were lower, compare to WKY. Our results support these findings. Both in liver and brain the quantity of GSH was significantly lower and the MDA level was significantly higher in SHR, compared to the control WKY (Table 2 and 3).

On the hepatic level, cytisine treatment did not affect the GSH quantity nor in SHR, neither in WKY, while the quantity of MDA was significantly increased in both strains. The lack of toxicity on GSH might be due to the lack of bioactivation of cytisine to metabolites that might conugate with GSH and cause its depletion. On the other hand, on the basis of our results we could suggest that the entire molecule of cytisine might have the ability to induce a process of lipid peroxidation, manifestated by the observed increase in MDA quantity.

There are a number of *in vivo* studies on the potential hepatotoxic effect of cytisine in different animal species. The study of Angelova (1971) determined that chronic administration of cytisine to rats, at a dose of 1.35 mg/kg during 90 days caused a 2-fold increase in blood glutamate pyruvate transaminase (GPT) concentration, without significant changes in blood glutamic oxaloacetic transferase (GOT) and alkaline phosphatase. Such changes were not observed when cytisine was administered during 45 days to mice (3.3 mg/kg) and 180 days to rats (0.45 and 0.9 mg/kg) or dogs (0.46 mg/kg). In our experiment cytisine, administered orally 5 mg/kg for 14 days did not significantly change the values of serum transaminase activity, ASAT and ALAT, in any of the treated strains. These results might be due to the shorter period of administration (14 days) and to the large individual variations.

On the brain level, multiple cytisine administration caused more prominent toxicity in SHRs, resulted in GSH depletion and increased MDA quantity, while in WKY strain did not exert any toxic effect. Reavill *et al*. (1990), in their studies in rats found out that cytisine crosses the blood-brain barrier less readily than nicotine. This might be one of the possible explanations for the observed lack of brain toxicity in WKY rats. On the other hand, it is proved that in chronic hypertension the blood brain barrier is characterised with an increased permeability due to disrupted tight junctions caused by endothelial dysfunctions (Lippoldt *et al*., 2000). The better permeability of cytisine through the BBB in hypertensive rats, slower blood brain circulation in this state (Kishi *et al*., 2004) could explain the higher brain toxicity of cytisine in hypertensive animals.

In conclusion, the results of our study suggest higher brain toxicity of cytisine in spontaneously hypertensive rats, that might be due to their pathophysiological characteristics.
